# The Michigan Model of Infant Mental Health Home Visiting increases preventative services while decreasing emergency services for children

**DOI:** 10.3389/fpsyg.2025.1549246

**Published:** 2025-04-11

**Authors:** Jerrica Pitzen, Danielle Rice, Barbara Durán, Jennifer Jester, Jessica Riggs, Megan Julian, Brendan Appold, Maria Muzik, Katherine Rosenblum, Michigan Collaborative for Infant Mental Health, Emily Alfafara, Carla Barron, Holly E. Brophy-Herb, Nora L. Erickson, Hiram E. Fitzgerald, Alissa C. Huth-Bocks, Meriam Issa, Jennifer M. Jester, Megan M. Julian, Jamie M. Lawler, Rena Menke, Alyssa S. Meuwissen, Alison L. Miller, Maria Muzik, Larissa N. Niec, Jerrica Pitzen, Julie Ribaudo, Jessica Riggs, Katherine L. Rosenblum, Sarah E. Shea, Paul Spicer, Ann M. Stacks, Chioma Torres, Laurie Van Egeren, Rachel Waddell, Christopher L. Watson, Deborah J. Weatherston, Kristyn VanDahm

**Affiliations:** ^1^Michigan Medicine, University of Michigan, Ann Arbor, MI, United States; ^2^School of Social Work, Wayne State University, Detroit, MI, United States; ^3^School of Social Work, University of Michigan, Ann Arbor, MI, United States; ^4^Department of Psychiatry, University of Vermont, Burlington, VT, United States

**Keywords:** healthcare utilization, referral rates, Infant Mental Health, well child visits, early intervention

## Abstract

**Objective:**

This study examined the impact of a relationship-based intervention, the Michigan Model of Infant Mental Health Home Visiting (IMH-HV), on infant/child referrals and receipt of physical health services.

**Method:**

Using a randomized controlled trial (RCT) design, participants included community-recruited mother-infant/toddler dyads who were randomized to treatment (IMH-HV) or control. Participant-reported healthcare, related service referrals received, and number of medical visits attended at baseline, 6-, and 12-month were examined.

**Results:**

Families assigned to IMH-HV were more likely to receive (OR = 13.6, *p* = 0.001) and follow up on referrals (OR = 7.1, *p* = 0.00), and found them more helpful than the control group (OR = 3.9, *p* = 0.03). Children in the treatment group received services in the emergency department (ED; 14.7%) less often compared to control group (34.4%). At 12 months, control group children were more likely to miss well-child visits compared to the IMH-HV group.

**Conclusion:**

These results demonstrate that families who receive IMH-HV services increase their access to and utilization of resources to reduce the impact of some of the most harmful social determinants of poor health, developmental, and relational outcomes. Unique components of IMH-HV that might explain this include attending to concrete needs, referrals for medical care, and providing developmental guidance.

## 1 Introduction

Pediatric preventive care is vital to the development of infants and toddlers. Well-child visits are key to pediatric preventive care, and families typically receive referrals for needed services at these visits. Other aspects of preventive care, such as developmental assessments and early intervention, promote positive health behaviors and reduce adverse health outcomes in infants and toddlers. Children who attend well-child visits are more likely to be up to date on their immunizations (Freed et al., 1999) and attend preventive dental visits sooner (Chi et al., 2013; Tiwari et al., 2019). The benefits of well-child visit attendance may extend into adulthood, as access to well-child visits in infancy is associated with more positive health, education, and economic outcomes by age 40. However, many families experience significant and varied barriers to accessing well-child visits and may be at heightened risk for missed developmental concerns, health problems, contraction of dangerous transmittable disease, and more. Cross-disciplinary efforts are underway to develop interventions targeting the increase of health care utilization for children and families. In the present study, we present findings from a randomized control trial (RCT) that support the effectiveness of the Michigan Model for Infant Mental Health-Home Visiting in increasing health care utilization in a sample of mother-infant dyads.

Missed well-child visits have important health and wellness implications for young children and may set the stage for poor healthcare utilization in the future. Research on associations between missed well-child visits and overreliance on other forms of healthcare (e.g., emergency departments, or urgent care) is mixed. Children with chronic health conditions or medical complexity were more likely to be hospitalized when their well-child visit attendance was lower (Shumskiy et al., 2018; Tom et al., 2010). Lawson et al. (2017) found no relationship between the number of well-child visits and the number of emergency department visits in the first 2 years of life. Regarding urgent care use, Burns et al. (2020) found children who relied heavily on urgent care centers had fewer visits with their primary care provider (PCP), including well-child visits, than children with low urgent care reliance; however, Montalbano et al. (2017) found that, among a pediatric Medicaid population, children who attended urgent care centers had higher healthcare utilization overall, with greater attendance at well-child visits and higher emergency department utilization.

While many parents acknowledge the importance of well-child visits for their child’s health, several barriers exist that make it difficult for parents to regularly attend these and other forms of preventive care. Working parents, those who lacked transportation, and those with a high number of depressive symptoms were more likely to miss well-child visits (Jhanjee et al., 2004). Additionally, mothers with no insurance or public health insurance, more children, or experiences of intimate partner violence were more likely to miss well-child or prenatal visits (Wolf et al., 2021). Interventions to increase healthcare utilization during infancy and toddlerhood should therefore focus not only on logistical and financial barriers, but also address other needs of parents, such as mental health and physical safety, all of which have the potential for downstream impacts on children’s access to preventive healthcare.

Home visiting programs provide a unique model of intervention that may help to reduce or eliminate significant barriers parents face in accessing healthcare for their children. Many of these programs aim to improve child health as a primary treatment goal and have demonstrated success in increasing the number of well-child and dental visits families attend (Avellar and Supplee, 2013; Fergusson et al., 2005). However, these programs differ in their structure, intensity, and approach to addressing healthcare access. The Michigan Model of Infant Mental Health Home Visiting (IMH-HV) is a needs-driven dyadic intervention aimed at promoting positive relationships between the parent and child, integrating case management, mental health support, and concrete assistance to address social determinants of health. Unlike other home visiting models, helping parents gain access to material needs, childcare, and healthcare is a core component of the IMH-HV model, in addition to providing emotional support, developmental guidance, increasing life coping skills and social supports, and utilizing Infant-Parent Psychotherapy (Weatherston et al., 2020). This dual emphasis on relational and practical support may enhance the intervention’s effectiveness in increasing well-child visit attendance and improving healthcare access, over and above other models of home-visiting.

Several other home visiting models have aimed to improve families’ engagement with health care. Child First pairs mental health clinicians with care coordinators to provide relationship-based psychotherapy and link families to community services, effectively increasing access to medical services (Lowell et al., 2011). Healthy Families America provides weekly home visits for 6 months, promoting well-child visit adherence and maternal health service use (LeCroy and Lopez, 2020). The Maternal Infant Health Outreach Worker (MIHOW) program, utilizing peer mentors to provide monthly visits with parenting education and referrals, increased families’ connections to resources by 6 months postpartum (Lutenbacher et al., 2018). Family Connects employs nurse home visits that enhance community connections, though visits are not weekly (Dodge et al., 2013a,b). The MOM Program offers brief home visits aligned with well-child visits to increase early intervention referrals and provide health education (Schwarz et al., 2012). SafeCare Augmented combines parenting skills training with motivational interviewing and risk factor screening, resulting in more referrals and linkages to services (Silovsky et al., 2011). In contrast, Early Head Start’s home-based option provides comprehensive child development services but has shown limited impact on families’ health service access (Love et al., 2001, 2002). Of these programs, only Child First, MOM Program, and Early Head Start effectively increased linkages to early intervention services.

The Michigan Model of Infant Mental Health Home Visiting expands the home visiting literature by offering a model with more intensive, frequent, and sustained intervention, typically spanning multiple years. Unlike many programs that primarily target either parenting skills or health service linkages, the Michigan Model integrates a comprehensive, relationship-based approach addressing maternal mental health, parenting, child development, and social determinants of health. Home visitors, who are master’s level clinicians, receive specialized training in infant mental health, reflective supervision, and trauma-informed care. The model’s depth and breadth of services—along with its flexible, individualized approach—position it as a uniquely holistic intervention capable of addressing both maternal well-being and child outcomes within the context of vulnerable families’ lives.

To increase access to healthcare, the IMH-HV home visitor serves as a case manager to identify the needs of each family and refer resources accordingly (Weatherston and Tableman, 2015). Support may be material in nature—such as food or clothing—or less tangible to facilitate the wellbeing of the parent and child—such as referring caregivers to their own mental health services, helping caregivers access public assistance, or connecting families to childcare services, developmental assessments, or healthcare. Partnerships between healthcare providers and home visitors may enhance the efficacy of home visiting programs for improving child health outcomes (Avellar and Supplee, 2013). IMH-HV home visitors are encouraged to develop working relationships with physicians and nurses to support caregivers around building their own relationships with healthcare providers via a ‘warm handoff.’ Further, IMH-HV home visitors may gain permission to communicate with healthcare providers to ensure immediate concerns regarding the child or caregiver’s health are addressed and to support parents in understanding instructions around medication use and compliance (Weatherston and Tableman, 2015). IMH home visitors also address barriers to attending appointments by helping caregivers access transportation via the provision of vouchers, identifying support people who can transport families, or by working with volunteer organization(s) that provide transportation to healthcare appointments (Weatherston and Tableman, 2015).

The goal of the current study is to examine the ways in which IMH-HV may contribute to increases in preventive healthcare utilization via a randomized controlled trial. Intervention group and control group families were compared on the number of referrals they were given for physical health or other services as well as their attendance at well-child and dental visits, their receipt of routine immunizations, and their use of other healthcare services such as urgent care centers or emergency departments.

## 2 Method

### 2.1 Study design

Data for the current study come from a larger, longitudinal study on the efficacy of IMH-HV. At present, IMH-HV services are delivered to eligible parent–child dyads across the state of Michigan. Services are available for eligible families from pregnancy through the first 3 years of a child’s life. For the current study, IMH-HV services were provided for up to 12 months. Families may self-refer or are referred by other providers (e.g., pediatrician, social worker). Participating families typically receive services in the home for 90–120 min on a weekly basis, with varied duration based on family need. IMH-HV providers have a masters-level degree in social work, psychology, or a related field. Training in the Michigan Model of Infant Mental Health Home Visiting (IMH-HV) requires participation in a year-long Learning Collaborative. IMH-HV RCT clinicians attended a standard 12-month Learning Collaborative with other community providers from the state community mental health system. The IMH-HV Learning Collaborative included 7 required in-person days across the first 7 months, weekly reflective supervision, and participation in bi-monthly case-based learning calls led by an IMH-E^®^ endorsed mentor. Didactic content for the in-person sessions covered each of the core components of the IMH-HV model, with a strong emphasis on infant-parent psychotherapy. Study clinicians held IMH-E^®^ endorsement as Infant Mental Health Specialists and met the standard training requirement of providing home-based IMH-HV services to at least two families with pregnant persons or parents of children <=24 months old. To ensure consistency in treatment delivery, providers completed the IMH-HV Treatment Fidelity Checklist after each treatment session with a participating family, tracking and adhering to at least one of the following broad IMH-HV strategies: (a) assessment, (b) connection to material needs, (c) addressing healthcare needs of the child, (d) addressing healthcare needs of the parent, (e) providing emotional/crisis support, (f) providing developmental guidance, (g) engaging in Infant-Parent Psychotherapy, (h) supportive life/goal planning, (i) fostering social support, (j) addressing special issues as they arise, (k) attending to safety plans, (l) planning for termination (Huth-Bocks et al., 2020). Forms were reviewed regularly with reflective supervisors. RCT study clinicians completed all required training activities with their Learning Collaborative cohort.

To qualify as a participant, mothers needed to be ≥18 years with legal custody of a child up to age 24 months (enrollment could occur during pregnancy). Exclusion criteria included symptoms of substance use disorders or psychosis. Importantly, participating mothers endorsed social, demographic, or psychological factors commensurate with those in the community who typically receive IMH-HV services. Only mothers who endorsed 2 or more of the following were eligible to participate: a screening score suggesting a possible diagnosis of depression, reported parenting challenges, eligibility for public benefits, and/or reporting 3 or more adverse childhood experiences (ACEs).

The current study utilized a randomized controlled trial design with *a priori* urn randomization procedures to ensure equal distribution of maternal ACEs, depression diagnosis, and income across treatment and control conditions. Those in the treatment condition were eligible to receive up to 12 months of IMH-HV services while those in the control condition did not receive services. This study received approval by the Institutional Review Board (ClinicalTrials.gov ID: NCT03175796, Michigan Medicine Institutional Review Board: HUM00124224).

### 2.2 Participants

For the current study, 66 mother-infant/toddler dyads participated in 5 waves of data collection. Assessments collected at baseline, 6- and 12-months were included in the present study. Retention rates overall were high, with 90.41% of participants completing the 12-month visit.

### 2.3 Measures

#### 2.3.1 Referrals for services

At the 6- and 12-month assessments, participants reported referrals they received in the prior 6 months. Referrals were defined as recommendations for a service by a provider or any other person. Caregiver services included those for physical health (e.g., doctor), mental health (e.g., therapy/medication), substance use treatment (e.g., counseling or Alcoholics/Narcotics Anonymous), public assistance (e.g., Medicaid), or tangible goods (e.g., diapers). Children’s services included mental/behavioral health services, physical health (e.g., immunizations), medical care for an illness or health need, developmental therapy/support (e.g., early intervention), or childcare/preschool (e.g., Head Start). For the present study, the total percentage of families receiving any referral reported was used. Perceived helpfulness of the referral is based on the percentage of caregivers who answered “yes” when asked if the service was helpful.

#### 2.3.2 Health care utilization

Information regarding health care utilization was obtained at all waves of data collection, with the current study using baseline, 6-, and 12-month assessment data. Participants were asked to indicate the type and number of medical visits that occurred for themselves and their child over the prior 6 months. Medical visits for the caregiver included visits to the emergency room, urgent care, and primary care visits, as well as annual exams and dental visits. Medical visits for the child included emergency room, urgent care, well-child, and dental visits, as well as visits to their pediatrician for an illness or injury. Participants also indicated whether their child received regular immunizations and if the caregiver and child have a primary health professional providing regular care. Since the American Academy of Pediatric Dentistry recommends that a child visit a dentist by 1 year of age, we only assessed dental visits for those children over 1 year of age.

The number of well-child visits at each assessment was compared to the number of visits recommended by the American Academy of Pediatrics, based on the age of the child (Hagan et al., 2017). To capture the entire course of treatment, the number of well-child visits reported for the prior 6 months both at the 6-month assessment and the 12-month assessment were summed for a total number of visits in the prior year. A ratio of total visits to recommended visits for each time was created. This was then dichotomized into “on schedule” for those attending more than half of the recommended visits and “behind schedule” for those reportedly attending equal to or fewer than half of the recommended visits.

### 2.4 Data analysis

#### 2.4.1 Frequentist logistic regression

For referral data, logistic regression was used to predict receiving any referral, for child and for caregiver, with treatment group as a predictor. When significant differences (*p* < 0.05) were found for “any referral,” different categories of referrals were examined to find the specific type of referral that differed between treatment and control groups.

For health care data, logistic regression was used to predict the binary variables for: (a) being behind schedule with well child visits, and (b) any emergency department (ED) visit. Potential covariates were examined, including demographics, perinatal health issues such as being in neonatal intensive care or birth complications, and baseline values of corresponding binary variables (baseline ED visits and baseline “behind schedule” for well child visits). Any covariates correlated (*p* < 0.1) with the outcome were included in the first logistic regression models. Models were then estimated sequentially, and predictors were removed in the next model if the predictor weight had a significance value >0.1. Logistic regression was also used to examine the difference between treatment and control groups in receiving referrals, following up and finding them helpful and for other health services, such as routine immunizations for children. The Firth penalized likelihood method was used in cases of quasi-complete separation of points in the logistic regression model (Firth, 1993; Heinze, 2006).

#### 2.4.2 Missing data

Missing data were multiply imputed using PROC MI in SAS 9.4. The imputation included variables that were correlated with outcome variables or with missingness at *p* = 0.1 or lower. Fully conditional specification (FCS) was used to create 40 imputations, using discriminant function for the binary variables (Berglund, 2015). Trace plots were visually examined and showed no apparent trends for any of the variables. Logistic models were run for each imputation and results combined using PROC MIANALYZE.

#### 2.4.3 Bayesian analysis

To incorporate data from prior published studies regarding the effect of home visiting treatment on ED use, we conducted a Bayesian analysis for the prediction of ED visits (Kaplan, 2024; de Leeuw and Klugkist, 2012). This analysis allowed us to formally introduce data (i.e., sign and size of parameters) into our model from earlier studies that examined health care utilization in trials with similar treatments. This type of analysis is particularly useful in studies such as ours which have a relatively small sample size. Effects of treatment found in the current study are combined with effects found in prior studies to increase the confidence in the results.

This analysis was performed using PROC BGLIMM in SAS 9.4. The prior distribution of the parameter estimate for the treatment effect was assumed to be a normal distribution with the mean and variance based on three selected published studies. Parameter estimates and their variances from the three studies were averaged. The average of the parameter estimates was used as the mean of the normal distribution and 10 times the range of the three estimates was used as the variance.

## 3 Results

See Table 1 for demographic information. At baseline, children were 12.03 months old (*SD* = 6.62). Seven women were pregnant at baseline. Overall, there was high economic need across the sample, as evidenced by enrollment in Medicaid insurance, and relatively low family income. Children in the study were predominantly White (78.46%) or Black (32.31%); multiple race/ethnicity descriptors could be chosen.

**Table 1:** Sample demographics.

	Total sample (66)		Treatment sample (33)		Control sample (33)	
	Range	*M* (*SD*)	Range	*M* (*SD*)	Range	*M* (*SD*)
Maternal age (years)	22–44	32.45 (5.46)	22–44	33.08 (5.04)	22–42	31.82 (5.86)
Child age (months)	0–24	12.03 (6.62)	0–24	12.76 (6.90)	0–24	11.12 (6.21)
			
	% (*n*)		% (*n*)		% (*n*)	
Family income variables						
Household income <$20,000	18 (27.27%)		10 (30.30%)		8 (24.24%)	
Household income >$20,000	48 (72.73%)		23 (69.70%)		25 (75.76%)	
Currently receive Medicaid	30 (45.45%)		15 (45.45%)		15 (45.45%)	
Child race/ethnicity						
White	51 (78.46%)		27 (84.38%)		24 (72.73%)	
Black	21 (32.31%)		10 (31.25%)		11 (33.33%)	
Hispanic or Latino/a	9 (13.85%)		7 (21.88%)		2 (6.06%)	
Other/Not Specified	9 (13.85%)		3 (9.09%)		6 (18.18%)	

The only statistically significant difference between Treatment and Control groups was that the Treatment group had significantly more Hispanic or Latino/a children than the Control group. Participants could choose as many race/ethnicity indicators as needed to describe their child’s race/ethnicity.

### 3.1 Referrals

We examined differences across treatment groups in whether the family received any referral for the caregiver or child at 6 and 12 months. Importantly, at baseline prior to randomization or receipt of treatment, there were no differences in referrals received between the two groups.

**Figure 1:**
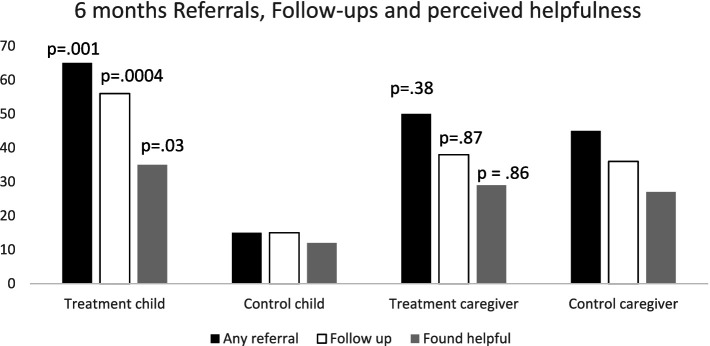
Percentage of families receiving any referral for child or caregiver, following up on referral and perceived helpfulness, by treatment group, at 6-month time point. (*p* values indicate significance of differences between treatment and control groups).

At 6 months, 65% of families in the treatment group were given referrals for their child, whereas only 15% of those in the control group received referrals for their child (OR = 10.27 *p* < 0.0001). Comparatively, 50% of caregivers in the treatment group were given referrals for themselves compared to 45% of caregivers in control group (OR = 1.44, *p* = 0.46). At 12 months, there was no difference between the groups in probability of families receiving referrals for children (OR = 1.05, *p* = 0.92) or for caregivers (56% treatment vs. 47% control, OR = 1.44, *p* = 0.46). Figure 1 shows the number of families who received any referrals for caregiver or for child, grouped by treatment group at 6-months.

Additionally, Figure 1 shows the number of families who followed up on a referral received for their child at the 6-month time point. Both groups were very likely to follow up on referrals, *if they received them*. Those families in treatment were more likely to *both* receive and follow up on a referral (OR = 7.1, *p* = 0.0004) than families in the control group. For the control families, 15% received and followed up on the referral, but for treatment families, 56% received and followed up on a referral. Furthermore, treatment families were more likely to find the referrals given to them for their children to be helpful (OR = 3.9, *p* = 0.03) than control families.

After examining overall referrals differences across treatment groups, types of different referrals received by the treatment and control groups for children at 6 months were examined. Referrals related to physical health were received at a somewhat higher rate in the treatment group (OR = 1.3, *p* = 0.075), and referrals for early intervention were significantly higher in the treatment group (OR = 2.2, *p* = 0.005) compared to control. No other category of referrals was different between groups.

### 3.2 Well-child visits

**Figure 2:**
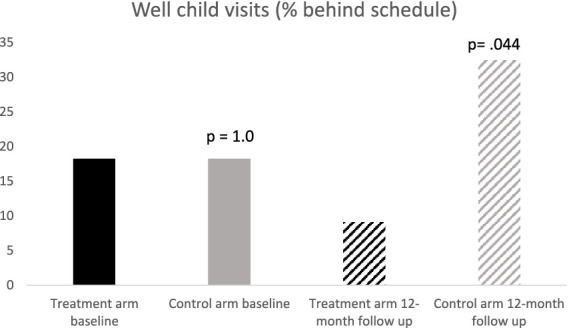
Percent of sample who had attended less than half of recommended well-child visits (*p* values indicate significance of differences between treatment and control groups).

The number of well-child visits at each assessment was compared to the number of visits recommended by the American Academy of Pediatrics, based on the age of the child. Figure 2 shows percentage “behind schedule” for well-child visits at baseline and 12-month follow up.

Treatment and control groups had similar rates of being behind schedule at baseline (treatment = 16%, control = 17%, OR = 1.10, *p* = 0.88). In contrast, the treatment group decreased the behind schedule rate to 14.7% in the year of treatment, whereas the control group increased to 32.4% in the same year (OR = 0.26, *p* = 0.044).

Logistic regression was used to model “behind schedule” for well-child visits. The final model contained only the treatment group as a predictor. The odds ratio for the treatment group predicting “behind schedule” was 0.26 (*p* = 0.044), indicating that those in the treatment group had lower odds of being behind schedule for well child visits than the control group.

### 3.3 Routine immunizations, urgent care, dental visits

As shown in Table 2, at baseline, 92% of the sample reported that their child had routine immunizations (97% treatment group and 88% control group). At the 12-month follow-up, results were similar, with 91% of the treatment group and 81% of the control group reporting the child having routine immunizations, with little difference between the two groups (OR = 2.2, *p* = 0.27).

**Table 2:** Health care utilization in treatment and control arms for baseline and 12-month follow up.

	Treatment arm baseline	Control arm baseline	Treatment arm 12-month follow up	Control arm 12-month follow up
Routine immunizations (% yes)	97.0%	88.8%	91.2%	81.3%
Urgent care visits (% any visit)	15.2%	3.0%	11.8%	9.4%
Dentist (any dentist visit)	15.2%	24.2%	36.0%	52.4%

Relatively few caregivers endorsed the use of urgent care for their child. At baseline, only 6 children (8.2%, 5 in treatment group and 1 in control group) were reported to have had an urgent care visit in the prior 6 months. At the 12 month follow up, only 7 children (9.6%, 4 treatment group and 3 control group) were reported to have used urgent care in the prior 6 months. Groups did not differ on urgent care use (treatment group OR = 1.3, *p* = 0.78; see Table 2).

Mothers were asked if they had taken their child to a dentist at each time point. When examining the percent of children with any dentist visit, the treatment group showed a slightly lower rate of dentist visits (OR = 0.53, *p* = 0.29). Both groups increased over the course of the study, but there was not a significant difference (see Table 2).

### 3.4 Emergency department (ED) visits

**Figure 3:**
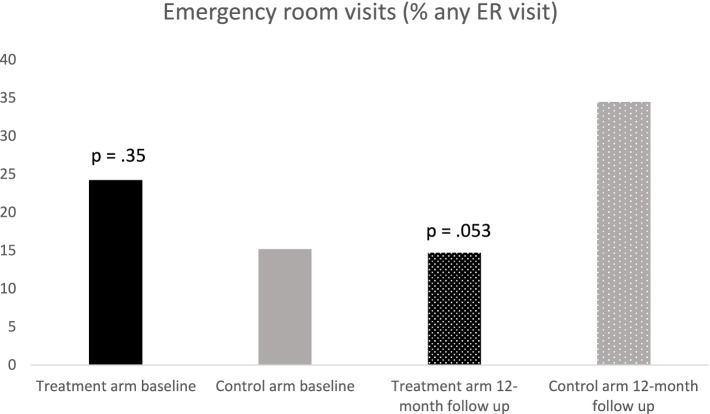
Percent of sample endorsing any ER visit in 6 months prior to the assessment.

As seen in Figure 3, treatment and control groups did not differ significantly in ED use at baseline (chi-square = 0.86, *p* = 0.35), with 24.2% of children in the treatment group having ≥1 ED visit compared to 15.2% of the control group. At the 12-month follow-up, only 14.7% of children in the treatment group reportedly had an ED visit within the last 6 months. In contrast, of the control group children, 34.4% reportedly had at least one ED visit.

For the frequentist perspective, logistic regression was used to examine the effect of treatment group on having at least one ED visit at 12 months, following the steps outlined in the data analysis section. The final model included treatment condition and child age as predictors. The odds ratio for the effect of the treatment group was 0.29 (*p* = 0.053). The analysis was repeated using Bayesian analysis with informative priors based on three published studies (Dodge et al., 2013a,b; Kilburn and Cannon, 2017; Olds et al., 1994). The prior distribution was normal (mean = −0.85, variance = 6.4). The mean of the posterior distribution for the treatment effect was –1.4 and the highest posterior density interval, converted to odds ratio was 0.067, *p* = 0.89. This indicates that, considering the current study and past results from the literature, the treatment group was less likely to have an ED visit in the prior 12 months compared to the control group. The fact that the highest posterior density interval does not contain “1” indicates confidence in this result. Compared to the frequentist logistic regression results, the mean OR of 0.25 was very close to the OR of 0.29 from the frequentist analysis and the credible interval was slightly smaller, due to the effects of prior knowledge from the literature.

## 4 Discussion

Given the important impact of children’s accessing preventive healthcare services on several long-term positive outcomes (Freed et al., 1999), the goal of this paper was to examine the effects of the Michigan Model of IMH-HV treatment on children’s healthcare utilization and families’ use, and perceived helpfulness, of referrals for physical health and other services.

Regarding referrals, although both the treatment and control groups had opportunities over the course of the study to receive referrals for services, and while both the treatment and control families were equally likely to follow up on a referral received, those in the treatment group were much more likely to receive a referral for a service. This meant that a larger percentage of families in the treatment condition received, followed-up on, and reported finding a referral helpful. Additionally, those in the treatment group received more referrals for physical health or early intervention services. These results lend support to the importance of parents having trusting relationships with their IMH-HV clinicians; to be comfortable having discussions regarding their child’s health and development and to acknowledge if their child is struggling in one or more areas. Trust between provider and parents is central to the IMH-HV model and is cultivated by providing consistent, present, compassionate, and responsive care, connecting families to resources (e.g., food, formula, housing, healthcare, childcare, and more) while providing emotional support in the context of stressors. Further, IMH-HV providers are trained in cultural sensitivity, centering the families’ beliefs, attitudes, and values while acknowledging their own biases in relation to treatment through reflective supervision (Weatherston et al., 2020). IMH-HV clinicians perform routine developmental assessments that can inform the caregiver if their infant/toddler is developing as expected or if further assessment is necessary. Moreover, the presence of a trusting relationship between caregivers and clinicians allows the IMH-HV clinician to provide referrals for services and for the caregiver to trust that these services have the potential to benefit their child. In addition to referring families for services, clinicians help families navigate the referral process and coordinate with other professionals to best support the family. Given that treatment families not only received more referrals, but also were more likely to connect to care as a result of that referral, indicates that the approach used in IMH-HV may be particularly successful in preventing health issues and developmental issues via early intervention.

Consistent with other home visiting programs (Fergusson et al., 2005), those in the treatment group were less likely to be behind schedule on their well-child visits by 12 months than those in the control group, with control group families growing further behind over the course of the study. However, there was no difference in the percentage of treatment group or control group children on receipt of routine immunizations. Families receiving IMH-HV services may learn about the added benefits of well-child visits beyond receiving immunizations, such as discussing developmental milestones, screening for other health concerns, or receiving support with sleep, feeding, or eating concerns. Further, IMH-HV clinicians work to minimize barriers regarding attendance of well-child visits, which may help parents stay on schedule.

Treatment and control group families were not different regarding urgent care usage over the course of the study; however, very few families overall reported utilizing urgent care. Significant differences were found for emergency department utilization, with treatment group families being less likely to visit the ED over the course of 12 months. Those in the treatment group may have developed a stronger relationship with their child’s PCP due to their increased well-child visit attendance, thereby learning that, in certain non-emergent cases, contacting their PCP may be a better option than taking their child to an ED. Further, the increased attendance of well-child visits may contribute to increased detection of chronic illnesses associated with greater reliance on the ED over time (Tom et al., 2010).

Notably, evidence from both the present study and the broader home-visiting literature supports the effectiveness of the Michigan Model of IMH-HV in racially, ethnically, and socioeconomically diverse communities. Families from marginalized backgrounds—particularly Black and Hispanic families—experience significantly lower rates of well-child visit attendance compared to White families, with barriers to accessing care ranging from structural obstacles like transportation to experiential challenges such as racial/ethnic discrimination and negative interactions with healthcare providers (Abdus and Selden, 2024; Fahey et al., 2024). These disparities underscore the need for interventions that address both practical and relational barriers to healthcare utilization. The Michigan Model of IMH-HV is designed to meet this need through its unique combination of tangible, case management support and relationship-based services that are intentionally adaptable to the cultural and socioeconomic contexts of the families it serves.

Although the present study sample comprises approximately 75% White and 25% Black participants—closely mirroring Michigan’s demographic composition (U.S. Census Bureau, 2021)—its socioeconomic makeup may not fully reflect the broader population of families served in community-based IMH-HV programs. However, prior research demonstrates that the Michigan Model of IMH-HV is effective with families from a range of marginalized backgrounds, including those with lower income, lower educational attainment, and higher psychosocial risk (Huth-Bocks et al., 2020). A core tenet of the model is its emphasis on cultural humility, responsiveness, and individualization, with home visitors trained to integrate families’ cultural values, beliefs, and experiences into service delivery (Weatherston et al., 2020). Simultaneously, other home visiting models, including Child First, Early Head Start, and Family Connects, that also incorporate a dual emphasis on relational and case management support, have similarly demonstrated success in improving healthcare utilization across diverse communities (Lowell et al., 2011; Love et al., 2001, 2002; Dodge et al., 2013a,b). Taken together, these findings suggest that home-visiting programs such as the Michigan Model of IMH-HV that emphasize cultural sensitivity, relational approaches, and case management support have the potential to play a critical role in mitigating barriers to healthcare access among marginalized populations.

### 4.1 Limitations

This study, though promising, is not without limitations. Despite demonstrating good retention, these findings would be strengthened with data from a larger pool of families. This may be particularly needed when examining differences between treatment groups related to infrequently occurring events (e.g., use of urgent care). Additionally, this sample, although diverse in race and family income, was exclusively comprised of mothers and their children. This limits generalizability to diverse caregivers, including fathers, grandparents as parents, and foster parents. As IMH-HV services are routinely provided to these parent/caregivers in the community, future studies should examine health care access among a sample of diverse parent/caregiver types. Finally, this study relied on parent report; we did not review medical records to confirm parent-reported information about health care utilization. Future work may use multiple methods of data collection to increase validity of findings.

## 5 Conclusion

In conclusion, this study demonstrates the positive effect of the Michigan Model of IMH-HV on children’s access to preventive health care, including attendance at well-child visits, reduced reliance on emergency medical care, and increased receipt, follow-up, and satisfaction of referrals to specialty care. These findings support IMH-HV as a home visiting program which is responsive and adaptive to family needs, seemingly addressing many barriers to care connection cited in the research, including social determinants of health, and parent mental health challenges.

## Data Availability

The raw data supporting the conclusions of this article will be made available by the authors, without undue reservation.

## Michigan Collaborative for Infant Mental Health Research

The Michigan Collaborative for Infant Mental Health Research (MCIMHR) is composed of researchers from eight universities and from the Alliance for the Advancement of Infant Mental Health, whose efforts are aligned with the promotion of research of Infant Mental Health. Core MCIMHR members have collaborated in the design, implementation and/or dissemination of the current study. MCIMHR core members include (in alphabetical order): Emily Alfafara, Carla Barron, Holly E. Brophy-Herb, Nora L. Erickson, Hiram E. Fitzgerald, Alissa C. Huth-Bocks, Meriam Issa, Jennifer M. Jester, Megan M. Julian, Jamie M. Lawler, Rena Menke, Alyssa S. Meuwissen, Alison L. Miller, Maria Muzik, Larissa N. Niec, Jerrica Pitzen, Julie Ribaudo, Jessica Riggs, Katherine L. Rosenblum, Sarah E. Shea, Paul Spicer, Ann M. Stacks, Chioma Torres, Laurie Van Egeren, Rachel Waddell, Christopher L. Watson, Deborah J. Weatherston, and Kristyn VanDahm.

## References

[ref1] Abdus S. Selden T. M. (2024). Racial and ethnic disparities in attendance to well-child visit recommendations during COVID-19. Acad. Pediatr. 24, 922–929. doi: 10.1016/j.acap.2024.04.003, PMID: 38614214

[ref2] Avellar S. A. Supplee L. H. (2013). Effectiveness of home visiting in improving child health and reducing child maltreatment. Pediatrics 132, S90–S99. doi: 10.1542/peds.2013-1021G, PMID: 24187128

[ref3] Berglund P. A. (2015). Multiple imputation using the fully conditional specification method: a comparison of SAS^®^, Stata, IVEware, and R. In Proceedings of the SAS Global Forum 2015 Conference. Cary, NC: SAS Institute Inc. American Academy of Pediatrics. 2081–2015.

[ref4] Burns R. R. Alpern E. R. Rodean J. Canares T. Lee B. R. Hall M.. (2020). Factors associated with urgent care reliance and outpatient health care use among children enrolled in Medicaid. JAMA Netw. Open 3, –e204185. doi: 10.1001/jamanetworkopen.2020.4185, PMID: 32374396 PMC7203605

[ref6] Chi D. L. Momany E. T. Jones M. P. Kuthy R. A. Askelson N. M. Wehby G. L.. (2013). Relationship between medical well baby visits and first dental examinations for young children in Medicaid. Am. J. Public Health 103, 347–354. doi: 10.2105/AJPH.2012.300899, PMID: 23237163 PMC3558774

[ref7] de Leeuw C. Klugkist I. (2012). Augmenting data with published results in Bayesian linear regression. Multivar. Behav. Res. 47, 369–391. doi: 10.1080/00273171.2012.673957, PMID: 26814603

[ref8] Dodge K. A. Goodman W. B. Murphy R. A. O’Donnell K. Sato J. (2013a). Randomized controlled trial of universal postnatal nurse home visiting: impact on emergency care. Pediatrics 132 Suppl 2, S140–S146. doi: 10.1542/peds.2013-1021M, PMID: 24187116 PMC3943376

[ref9] Dodge K. A. Goodman W. B. Murphy R. O’Donnell K. T. Sato J. M. (2013b). Toward population impact from home visiting. Zero to Three 33, 17–23, PMID: 23526864 PMC3606025

[ref10] Fahey N. Holt A. Cataltepe D. Brochier A. Stern A. Mazanec M.. (2024). Understanding barriers to well-child visit attendance among racial and ethnic minority parents. BMC Primary Care 25:196. doi: 10.1186/s12875-024-02442-0, PMID: 38831259 PMC11149240

[ref11] Fergusson D. M. Grant H. Horwood L. J. Ridder E. M. (2005). Randomized trial of the early start program of home visitation. Pediatrics 116, e803–e809. doi: 10.1542/peds.2005-0948, PMID: 16322138

[ref12] Firth D. (1993). Bias reduction of maximum likelihood estimates. Biometrika 80, 27–38. doi: 10.1093/biomet/80.1.27

[ref13] Freed G. L. Clark S. J. Pathman D. E. Schectman R. (1999). Influences on the receipt of well-child visits in the first two years of life. Pediatrics 103, 864–869. doi: 10.1542/peds.103.S1.864, PMID: 10103323

[ref14] Hagan J. F. Shaw J. S. Duncan P. M. (2017). Bright futures: Guidelines for health supervision of infants, children, and adolescents: pocket guide.

[ref15] Heinze G. (2006). A comparative investigation of methods for logistic regression with separated or nearly separated data. Stat. Med. 25, 4216–4226. doi: 10.1002/sim.2687, PMID: 16955543

[ref1900] Huth-Bocks A. C. Jester J. M. Stacks A. M. Muzik M. Rosenblum K. L. Michigan. (2020). Infant mental health home visiting therapists’ fidelity to the Michigan IMH‐HV model in community practice settings. Infant Ment. Health J. 41, 206–219., PMID: 32242965 10.1002/imhj.21839

[ref16] Jhanjee I. Saxeena D. Arora J. Gjerdingen D. K. (2004). Parents’ health and demographic characteristics predict noncompliance with well-child visits. J. Am. Board Fam. Pract. 17, 324–331. doi: 10.3122/jabfm.17.5.324, PMID: 15355945

[ref17] Kaplan D. (2024). Bayesian statistics for the social sciences. 2nd Edn. New York, NY: The Guilford Press.

[ref18] Kilburn M. R. Cannon J. S. (2017). Home visiting and use of infant health care: a randomized clinical trial. Pediatrics 139:e20161274. doi: 10.1542/peds.2016-1274 27980028

[ref19] Lawson N. R. Klein M. D. Ollberding N. J. Wurster Ovalle V. Beck A. F. (2017). The impact of infant well-child care compliance and social risks on emergency department utilization. Clin. Pediatr. 56, 920–927. doi: 10.1177/0009922817706145, PMID: 28438048

[ref20] LeCroy C. W. Lopez D. (2020). A randomized controlled trial of healthy families: 6-month and 1-year follow-up. Prev. Sci. 21, 25–35. doi: 10.1007/s11121-018-0931-4, PMID: 30039328

[ref2100] Love J. Kisker E. Ross C. Schochet P. Brooks-Gunn J. Boller K.. (2001). Building their futures: How Early Head Start programs are enhancing the lives of infants and toddlers in low-income families. Summary report. Report to Commissioner’s Office of Research and Evaluation, Head Start Bureau, Administration on Children, Youth and Families, and Department of Health and Human Services. (Princeton, NJ: Mathematica Policy Research).

[ref21] Love J. Kisker E. Ross C. M. Schochet P. Z. Brooks-Gunn J. Paulsell D.. (2002). “Making a difference in the lives of infants and toddlers and their families: the impacts of early head start” in Volumes I-III: Final technical report [and] appendixes [and] local contributions to understanding the programs and their impacts (Washington, DC: U.S. Department of Health and Human Services, Head Start Bureau).

[ref22] Lowell D. I. Carter A. S. Godoy L. Paulicin B. Briggs-Gowan M. J. (2011). A randomized controlled trial of child FIRST: a comprehensive home-based intervention translating research into early childhood practice. Child Dev. 82, 193–208. doi: 10.1111/j.1467-8624.2010.01550.x, PMID: 21291437

[ref23] Lutenbacher M. Elkins T. Dietrich M. S. Riggs A. (2018). The efficacy of using peer mentors to improve maternal and infant health outcomes in Hispanic families: findings from a randomized clinical trial. Matern. Child Health J. 22, 92–104. doi: 10.1007/s10995-018-2532-z, PMID: 29855840 PMC6153763

[ref24] Montalbano A. Rodean J. Canares T. Burns R. Lee B. Alpern E. R.. (2017). Urgent care utilization in the pediatric Medicaid population. J. Pediatr. 191, 238–243.e1. doi: 10.1016/j.jpeds.2017.08.035 29173313

[ref25] Olds D. L. Henderson C. R. Jr. Kitzman H. (1994). Does prenatal and infancy nurse home visitation have enduring effects on qualities of parental caregiving and child health at 25 to 50 months of life? Pediatrics 93, 89–98. doi: 10.1542/peds.93.1.89, PMID: 8265329

[ref26] Schwarz D. F. O’Sullivan A. L. Guinn J. Mautone J. A. Carlson E. C. Zhao H.. (2012). Promoting early intervention referral through a randomized controlled home-visiting program. J. Early Interv. 34, 20–39. doi: 10.1177/1053815112451849

[ref27] Shumskiy I. Richardson T. Brar S. Hall M. Cox J. Crofton C.. (2018). Well-child visits of Medicaid-insured children with medical complexity. J. Pediatr. 199, 223–230.e2. doi: 10.1016/j.jpeds.2018.04.003, PMID: 29752175

[ref28] Silovsky J. F. Bard D. Chaffin M. Hecht D. Burris L. Owora A.. (2011). Prevention of child maltreatment in high-risk rural families: a randomized clinical trial with child welfare outcomes. Child Youth Serv. Rev. 33, 1435–1444. doi: 10.1016/j.childyouth.2011.04.023

[ref29] Spielberger J. Burkhardt T. Winje C. Pacheco-Applegate A. Gitlow E. Carreon E.. (2021). Evaluation of the Illinois model of infant and early childhood mental health consultation pilot. Chicago, IL: Chapin Hall at the University of Chicago.

[ref30] Tiwari T. Rai N. Brow A. Tranby E. P. Boynes S. G. (2019). Association between medical well-child visits and dental preventive visits: a big data report. JDR Clin. Trans. Res. 4, 239–245. doi: 10.1177/2380084419841850, PMID: 31008682

[ref31] Tom J. O. Tseng C. W. Davis J. Solomon C. Zhou C. Mangione-Smith R. (2010). Missed well-child care visits, low continuity of care, and risk of ambulatory care–sensitive hospitalizations in young children. Arch. Pediatr. Adolesc. Med. 164, 1052–1058. doi: 10.1001/archpediatrics.2010.201, PMID: 21041598 PMC3551592

[ref32] U.S. Census Bureau (2021). Michigan: 2020 census. United States Census Bureau. Available online at: https://www.census.gov/library/stories/state-by-state/michigan-population-change-between-census-decade.html

[ref33] Weatherston D. J. Ribaudo J. Michigan Collaborative for Infant Mental Health Research (2020). The Michigan infant mental health home visiting model. Infant Ment. Health J. 41, 166–177. doi: 10.1002/imhj.21838, PMID: 32242955

[ref34] Weatherston D. Tableman B. (2015). Infant mental health home visiting: supporting competencies/reducing risks: manual for early attachments: IMH home visiting: Michigan Association for Infant Mental Health.

[ref35] Wolf E. R. Donahue E. Sabo R. T. Nelson B. B. Krist A. H. (2021). Barriers to attendance of prenatal and well-child visits. Acad. Pediatr. 21, 955–960. doi: 10.1016/j.acap.2020.11.025, PMID: 33279734 PMC8172669

